# Synchrotron structural biology at SSRL, the beginning and beyond

**DOI:** 10.1107/S160057752500788X

**Published:** 2026-01-01

**Authors:** Keith Hodgson

**Affiliations:** a Department of Chemistry and Stanford Synchrotron Radiation Lightsource Stanford University and SLAC National Accelerator Laboratory 2575 Sand Hill Road Stanford CA94305 USA; University of Manchester, United Kingdom

**Keywords:** pioneering synchrotron structural biology, SSRL, macromolecular crystallography, MAD phasing, technology developments, Stanford Synchrotron Radiation Project

## Abstract

Synchrotron X-ray structural biology has developed into a worldwide, highly productive enterprise whose capabilities are providing thousands of users annually with the means to make innovative and outstanding scientific discoveries based on biological structure and function, impacting areas of human health, bioenergy and sustainability and many others. This special virtual issue of *Journal of Synchrotron Radiation* (https://journals.iucr.org/special_issues/2025/ssrlprotein) includes a series of contributed papers from scientists at structural biology synchrotron centers worldwide that describe capabilities, scientific discoveries and future directions. In my article, I will start with a focus on the first macromolecular crystallography studies at SSRL that were published in 1976, followed by developments in anomalous scattering and multi-wavelength phasing. I will then highlight some technology and methodology developments including X-ray detectors and beamline automation, which were key in building robust, sustainable resources at SSRL and other synchrotron radiation facilities for the structural biology user community. I will highlight other related elements including accelerator R&D, consider some of the overarching factors which I believe have been important for the sustained success and growth of this enterprise for 50 years and counting, and highlight some of the indicators of the enormous success of this venture. Throughout I will comment upon some of the new developments and trends that are emerging.

## Abstract 2

It has been 50 years since the publication in *PNAS* of our first results on the utilization and demonstrated benefits of synchrotron radiation for macromolecular crystallography. In this brief article, I offer some personal observations and comments about that early research at Stanford and the Stanford Synchrotron Radiation Project (SSRP, later renamed SSRL), how it evolved, and consider key factors in how and why synchrotron radiation ultimately catalyzed a new and transformational paradigm for study of the structure of biomolecules.

## 1. Introduction

Let me begin on a personal note. My own scientific training at UC Berkeley and ETH Zürich included classic crystallography and its applications in inorganic chemistry, but not macromolecular crystallography (MX). Shortly after I joined the Stanford Chemistry faculty as an Assistant Professor in 1973, I became aware of the Stanford Synchrotron Radiation Project (SSRP). SSRP was an NSF-funded project that was building instruments to enable experiments to be done using synchrotron radiation (SR) from the SPEAR electron–positron colliding-beam storage ring at the Stanford Linear Accelerator Center (SLAC, later renamed the SLAC National Accelerator Laboratory). The SPEAR storage ring was operating for high energy physics experiments and the SR program was parasitic. Upon learning of the unique properties of synchrotron X-rays from Seb Doniach (then SSRP’s Director), it became immediately apparent to me that there could be impactful applications in structural biology and chemistry.

In addition to building up a standard small molecule X-ray laboratory and using synchrotron X-rays for X-ray absorption and EXAFS studies, we started an MX subgroup. We were fortunate to have Alex Wlodawer from UCLA and Marguerite Yevitz Bernheim from Penn State join as postdocs. A graduate student from Stanford’s Applied Physics department, James Phillips, also joined the group. About a year later, Julia Goodfellow came as a postdoc from UK. These talented young scientists and the proximity of our group in Chemistry on the Stanford main campus to SSRP at SLAC were key factors in enabling our pioneering studies reported in the 1976 *Proceedings of the National Academy of Sciences of the USA* (hereafter *PNAS*) paper (Phillips *et al.*, 1976 ▸), and subsequent work on anomalous scattering and multi-wavelength phasing (Phillips *et al.*, 1977 ▸). Another important related development during this period was meeting Lubert Stryer, who moved to Stanford from Yale to establish the new Department of Structural Biology in Stanford’s School of Medicine in 1976. Lubert was very keen on having X-ray crystallography be a part of the tools available at Stanford for structural studies of biomolecules and was exceptionally supportive. Roger Koeppe, a postdoc with Lubert Stryer, as well as Jeremy Berg, a Stanford Chemistry undergraduate, were also engaged in our relatively small but creative and highly motivated MX initiative at Stanford.

I was unaware of synchrotron radiation when I arrived at Stanford to begin my research program; the location of SSRP near the main campus was a completely serendipitous aid to my work. What was unique about SSRP (which was renamed SSRL in 1977) was that when it began operation in 1973 it was the first facility in the world to exploit the stability and brightness of a multi-GeV electron storage ring, SPEAR, to provide SR access to a broad spectral region, from VUV/soft X-ray to hard X-ray, for a large community of SR users. Storage rings, in which the particle energy is constant, are very stable sources of synchrotron light. In contrast, synchrotrons are circular particle accelerators where the particle energy varies with time as the magnetic field is changed and hence are quite unstable photon sources. Storage rings are the basis of all major SR light facilities in operation today (except for free-electron lasers). The primary function of SPEAR at that time was for high energy physics, with SSRP operating parasitically (this is often referred to as a ‘first generation synchrotron source’). Synchrotron X-rays from storage rings like SPEAR at SSRP were very bright compared with conventional X-ray sources, were ‘tunable’ in that they covered a wide photon energy range, were produced in precisely timed short pulses, and were quite stable. More details about the properties of synchrotron radiation available in the 1970s and its applications can be found in a number a review articles and texts, one focused on SSRL being *Synchrotron Radiation Research* edited by Herman Winick and Seb Doniach (Winick & Doniach, 1980 ▸).

It is important to stress that the benefits of synchrotron radiation for biological research, although not necessarily for MX, were being explored prior to our studies at SSRP. The first use of synchrotron X-rays to study a biological problem was performed by Kenneth Holmes and Gerd Rosenbaum in the early 1970s (Rosenbaum *et al.*, 1971 ▸) on the 7.5 GeV electron synchrotron at DESY in Hamburg, Germany. Their experiments were using X-ray fiber diffraction to investigate the structure of muscle. While studies such as those pursued by Holmes, Rosenbaum and colleagues were quite challenging to do when utilizing X-rays generated from a synchrotron versus those from a storage ring, these pioneering experiments clearly demonstrated the immense potential for applications of SR in structural biology. Learning of them certainly informed, encouraged and inspired many of us at Stanford, and more broadly in the scientific community, to vigorously pursue the use of SR for experiments in structural biology and chemistry.

The initial suite of five instruments (often referred to as beamlines, BL) constructed at SSRP in the early 1970s did not include one designed specifically for MX. There was, however, a beamline (BL1-4) designed and built for biological small angle X-ray scattering. This was a project led by John Baldeschwieler and colleagues from Caltech (Webb *et al.*, 1977 ▸). We realized that the BL1-4 endstation could be reconfigured to enable MX studies, primarily through the addition of a diffraction camera using film as the recording media. With the support of the SSRP technical staff and consent of Baldeschwieler and the Caltech team, we built up a temporary setup for carrying out the first X-ray MX studies at SSRP in late 1974, setting the stage for carrying out the experiments published in our landmark 1976 *PNAS* paper (Phillips *et al.*, 1976 ▸).

## 2. Pioneering structural biology developments and studies at SSRP/SSRL in the mid-1970s into the 1980s

### 2.1. MX and the 1976 *PNAS* paper

**Figure 1:**
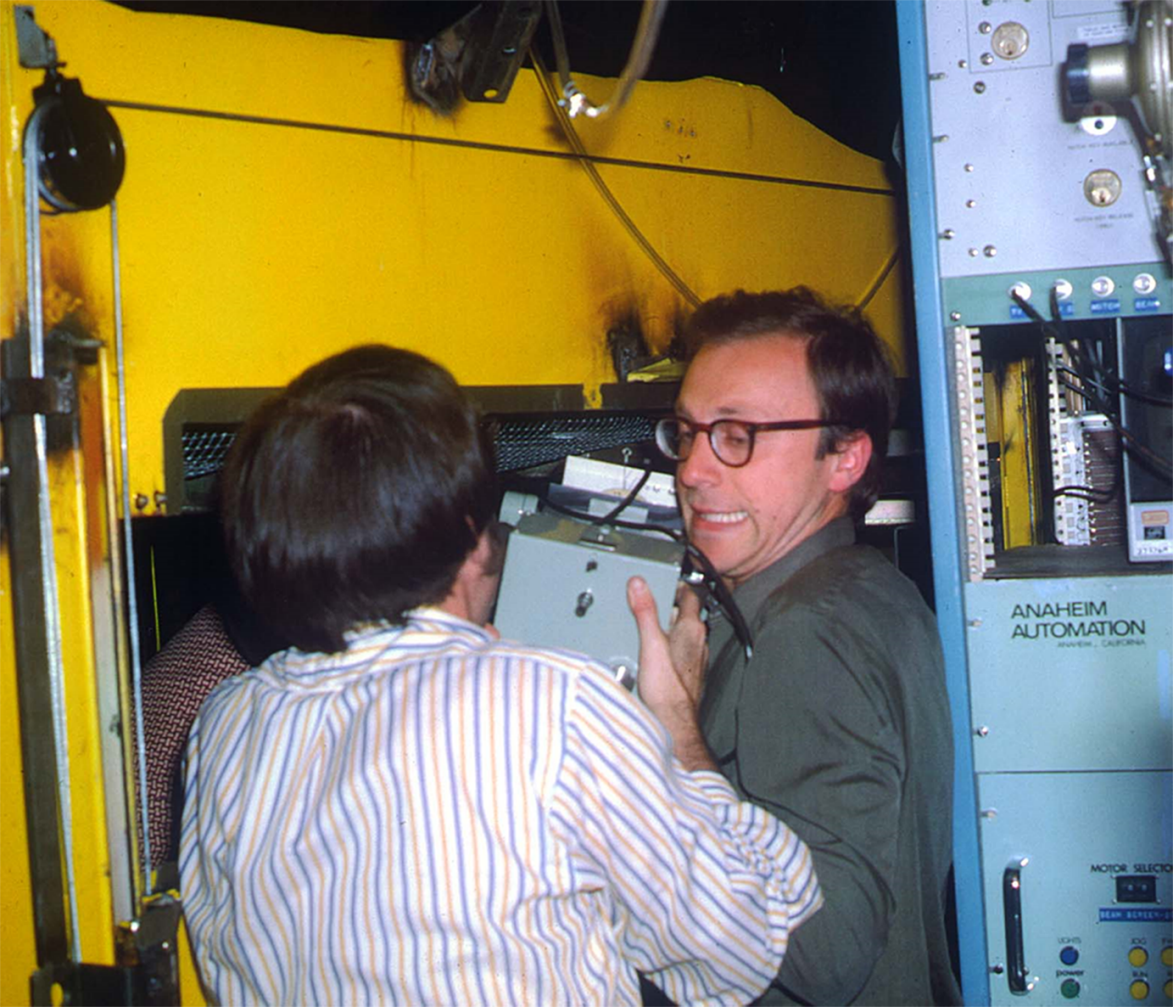
Keith Hodgson (back to camera) and Alex Wlodawer moving the Enraf-Nonius precession camera into the rear of the BL1-4 experimental hutch at SSRP, *ca* 1975.

The BL1-4 monochromator and mirror optical system and associated infrastructure designed and built by the Caltech group (Webb *et al.*, 1977 ▸) was modified by the inclusion of an Enraf-Nonius precession camera located in the downstream end of the experimental hutch/radiation enclosure. A standard pinhole collimator was used to restrict the final beam size. Given that the instrument and radiation enclosure were not intended for this purpose, it was indeed rather challenging to insert and align the precession camera as seen in Fig. 1 ▸.

With SSRP operating at the SPEAR storage ring in parasitic mode, a number of experiments were carried out as described in the *PNAS* paper (Phillips *et al.*, 1976 ▸). It was shown that single-crystal diffraction data could be measured from protein crystals with much shorter data collection times, around 60× faster than with a fine focus X-ray tube. We were able to successfully demonstrate the use of smaller crystals than typically used at that time for MX studies (*e.g.* ∼0.4 mm × 0.15 mm × 0.1 mm) and also were able to directly observe anomalous scattering effects of Fe (in rubredoxin) by selecting an X-ray wavelength close to the Fe *K* absorption edge. The effect of excellent collimation on the diffraction patterns was quite evident. We reached the overall striking conclusion that the much higher intensity and other qualities of SR (variable wavelength, collimation, high monochromaticity) offered significant advantages with regard to recording higher quality data to higher resolution and in shorter times than typically available from conventional sources.

These studies published in *PNAS* in 1976 have also been mentioned in the overview article for this issue (Helliwell *et al.*, 2025 ▸). Fig. 1 in that paper shows a photograph of the same experimental hutch on BL1-4, but with the four principal experimenters smiling at the camera. I would also note that Alex Wlodawer and colleagues published an interesting and informative review article on the impact of synchrotron radiation on MX (Dauter *et al.*, 2010 ▸) which includes a personal account of these pioneering experiments. Such was the beginning of the synchrotron revolution in MX at SSRP.

These first results began to rather quickly gain wide dissemination. For the UK, John Helliwell has described this (Helliwell *et al.*, 2025 ▸). In the US, meetings like those of the American Crystallographic Association (ACA) and the West Coast Protein Crystallography meetings at Warner Springs Ranch (Stroud, 2011 ▸) offered timely and ideal opportunities to discuss the results with the US protein crystallographic community and build a foundation that was important for expanding the capabilities on synchrotron sources for structural biology studies. I gave a talk about our preliminary results at the ACA meeting in 1975 in Charlottesville, Virginia, USA. At the Warner Springs meeting in 1976, I recall meeting several leading west coast protein crystallographers including David Eisenberg, Brian Matthews and Bob Stroud, and having vigorous discussions of the more detailed results and future potential. There was not always agreement that these initial results were more generally and broadly applicable, and where all this might lead, but it was an important beginning. Many of these early discussions and publications led to engagement of outstanding scientists and their research groups in the applications of SR in structural biology and started relationships institutionally that continued over many years, even through to today. Building these foundations was essential for the dramatic growth and impact of SR-enabled MX and the associated technical and methodological developments. These were key elements in terms of both scientific impact and gaining sustainable financial support for resource centers at the synchrotrons from government and private sources.

### 2.2. Parallel development of XAS/EXAFS techniques at SSRP for study of biological systems

In parallel with the developments in MX at SSRP, instrumentation for measuring X-ray absorption spectra and the related extended X-ray absorption fine structure (EXAFS) was being implemented on the neighboring beamline (BL1-5). X-ray absorption spectroscopy (XAS) methods are complimentary to crystallography when seeking structural information and the availability of high brightness X-rays spanning a wide energy range revolutionized the XAS technique and enabled a broad range of applications. The BL1-5 development was being led by the groups of Farrel Lytle (Boeing Company), Ed Stern and Dale Sayers (University of Washington), and a team from Bell Labs that included Peter Eisenberger, and in partnership with SSRP. Seb Doniach, who was the first director of SSRP, also had a keen interest in XAS. It was through discussions with Seb, and his graduate student Brian Kincaid, that I came to recognize the potential of using XAS and EXAFS for studying the electronic and metric structure of metal ion active sites in metalloproteins. An early accounting of these concepts as we saw them is found in the *PNAS* paper by Kincaid *et al.* (Kincaid *et al.*, 1975 ▸). We engaged in further developing and applying XAS/EXAFS to probe the structural chemistry of metals in a range of biological systems. It is important to note that, in parallel, the Bell Labs group that included Bob Shulman and collaborators also was investigating metalloproteins at SSRP using BL1-5, initially focusing on rubredoxin, a small redox protein containing Fe (Shulman *et al.*, 1975 ▸).

During the period 1976–1979, graduate students in my group, including Steve Cramer, Tom Eccles, Tom Tullius and collaborators, studied metalloproteins containing Fe, Cu and Mo in various metalloprotein active sites. We were able to make the first *de novo* determination of a fragment of the novel Mo–Fe–S metal cluster active site in nitro­genase (Cramer *et al.*, 1978*a* ▸), characterize the details of the key axial sulfur ligand to Fe in the active site of cytochrome P-450 (Cramer *et al.*, 1978*b* ▸) and identify an unusually short Cu—S bond in the ‘blue copper protein’ azurin (Tullius *et al.*, 1978 ▸). In a later review article published in 1997, we wrote ‘The promise of EXAFS as an important new tool to investigate the structure and function in biological and chemical systems was quickly realized’ (Doniach *et al.*, 1997 ▸).

My research sub-groups involved in synchrotron MX and in XAS/EXAFS interacted extensively and we were very aware of the phenomenon of anomalous scattering of metals in proteins and its relationship to the X-ray absorption phenomena. This further encouraged and motivated us to explore the topic of synchrotron-based anomalous scattering which we had first commented upon in the 1976 *PNAS* paper.

### 2.3. Anomalous dispersion – fundamentals and solving the phase problem

The availability of SR at SSRL for both single-crystal diffraction and XAS/EXAFS measurements stimulated a great deal of research interest in the well known phenomenon of anomalous X-ray scattering. While anomalous scattering at wavelengths away from absorption edges can be adequately described by atomic theory, large and rapid variations in anomalous scattering terms can and do occur near absorption edges, most notably for higher *Z* elements. The anomalous scattering terms, real (*f*′) and imaginary (*f*′′), can be experimentally determined as a function of wavelength, or in principle calculated where there are adequate applicable molecular theories.

We and others carried out detailed systematic studies of anomalous scattering behavior near absorption edges of transition metals, lanthanides and actinides at SSRL (hereafter I will use SSRL versus SSRP as much of this work was done after 1977). For example, we measured very large anomalous scattering effects around the *L*-edges of the lanthanide ions. The so-called intense ‘white line’ features at *L*_3_ and *L*_2_ edges of the lanthanides are due to enhanced 2*p* → final *d* state transitions (versus 1*s* initial state for *K* edges). Values of *f*′′ could be directly determined from the X-ray absorption data, and those of *f*′ by a Kramers-Kronig dispersion relationship. Very large changes of the order of 20 electrons were seen in values of *f*′ and *f*′′ (Lye *et al.*, 1980 ▸). There were additional detailed studies in the early 1980s (*e.g.* for compounds of Cs, Co, Gd and Sm) by David and Lilo Templeton, Paul Phizackerley and Hodgson and collaborators (Templeton *et al.*, 1980 ▸; Templeton *et al.*, 1982 ▸). The Templetons also observed and measured X-ray dichroism near the*L*-edges of U using linearly polarized synchrotron X-rays (Templeton & Templeton, 1982 ▸).

Also of interest and potentially much greater impact was to use the large changes in anomalous dispersion terms observed around metal absorption edges to experimentally determine phases of structure factors. It was already known for example that changes in *f*′ affect the diffraction pattern in the same way as does the insertion of a heavy atom (Raman, 1959 ▸) and the concept of using two wavelengths to determine phases had been demonstrated using a conventional X-ray source (Hoppe & Jakubowski, 1975 ▸). Synchrotron radiation was seen as a potential game-changer for the applications of anomalous scattering for MX phasing.

Following up on the early work reported in our 1976 *PNAS* paper, we explored the significantly greater potential of synchrotron multiwavelength anomalous scattering to determine phases and calculate electron density maps. This was discussed in detail in two papers using data taken at SSRL (Phillips *et al.*, 1977 ▸; Phillips & Hodgson, 1980 ▸). Our first real demonstration of the power of synchrotron-based multiwavelength anomalous dispersion (MAD) phasing came through a collaboration with Hans Freeman and Mitch Guss (Guss *et al.*, 1988 ▸) where we were able to experimentally determine the phases and solve the structure of the basic ‘blue’ copper protein (CBP) from cucumbers. This was among the earliest successes in high resolution ‘*de novo*’ MAD phasing for solution of an unknown structure using SR. At the time of these experiments at SSRL, CBP crystals had been available for over a decade, yet the structure had defied solution by any other means. At SSRL, we collected MAD data at four wavelengths using a 2D multiwire area detector on BL1-5 (see Section 3 below) over a period of about eight days. Phasing of the 10 kD protein using the anomalous scattering from the native Cu atom gave an excellent electron density map at ∼2.5 Å resolution.

It is important to note that, around this same time, very significant innovations in MAD phasing were also being made by Wayne Hendrickson and his research group. One especially novel and subsequently widely adapted approach was the substitution of S-me­thio­nines by Se-me­thio­nines in proteins and using Se anomalous scattering to determine the phases. An accounting of this, relevant publications and related synchrotron crystallography research can be found in the excellent overview by Hendrickson (2000 ▸). It is completely clear that SR revolutionized the use of MAD phasing as its ‘tunability’ and extreme brightness provided for high quality measurements at any wavelength.

### 2.4. Evolution of the SSRL SMB Program and Center

For SR to become accepted and widely used by the biological scientific community, it was necessary to provide the means for effective access, training and user support for instruments located on the physically large synchrotron rings, often quite remote from the labs of the research groups. In the US, and indeed at many synchrotron facilities worldwide, the most successful and cost-efficient model that evolved was one of ‘centers of excellence’ where talented scientific and technical staff developed and maintained the instruments and operated them for access by the scientific user community. In the US, this approach formed the basis for what were the NIH-funded synchrotron structural biology centers (using the so-called P41 grant mechanism – more on this below in Section 5). The first NIH centers were funded at SSRL (1980) and Cornell/CHESS (1984) and were followed by several others later. The SSRL Structural Molecular Biology center, which continues operations to this day, featured synchrotron-based techniques of X-ray macromolecular crystallography, small angle X-ray scattering and XAS/EXAFS. Subsequently, in the US, structural biology center funding was also provided by the Department of Energy, Office of Biological and Environmental Research, typically in a manner coordinated with NIH funding where appropriate. This paradigm was a critical element in the success and growth of the synchrotron structural biology enterprise at SSRL and for other SR centers in the US.

The SSRL SMB program currently operates and supports four dedicated MX and one dedicated SAXS/WAXS beamlines. Two specialized beamlines are operated for conventional XAS spectroscopy, and an additional total of two beamlines for XAS imaging and advanced spectroscopic techniques like RIXS and HERFD provide capabilities within the SMB program. Details and specifications of these beamlines (as well as all others at SSRL) are found on the SSRL website (https://www-ssrl.slac.stanford.edu/ssrl/web/beam-lines/by-number).

## 3. Technology and methodology R&D for synchrotron MX studies

An essential and important element in the impact, growth and success of SR-enabled structural biology research at the large-scale user facilities has been the development of beamline instrumentation and methods that provided new, enhanced and more efficient means for making experimental measurements and analysis of data from biological systems. There are many relevant areas where progress has been remarkable, among them being in beamline optics, X-ray detectors, sample manipulation platforms, computing, robotics and remote access and others. While an extensive discussion is beyond the scope of the article, many excellent examples are found elsewhere in this issue. Here I will illustrate this with a few examples from our structural biology program at SSRL. I will also comment in Section 5 on some of the key synchrotron source developments enabled by advances in accelerator science that have also played a key role in the success and impact of the synchrotron structural biology enterprise.

### 3.1. Multiwire area detector system optimized for MAD MX studies

The earliest synchrotron diffraction and scattering studies used film as the recording medium. It was recognized by many in the community that significant gains could be made with improved X-ray detectors. Early developments focused on approaches such as adapting multi-wire detectors from high energy physics, the use of imaging plate technology developed by Kodak and Fuji and various image-intensifier based systems. CCD detector arrays, front ended by X-ray scintillators, were a very important advance and this was followed by what is today’s state-of-the-art pixel array detectors.

**Figure 2:**
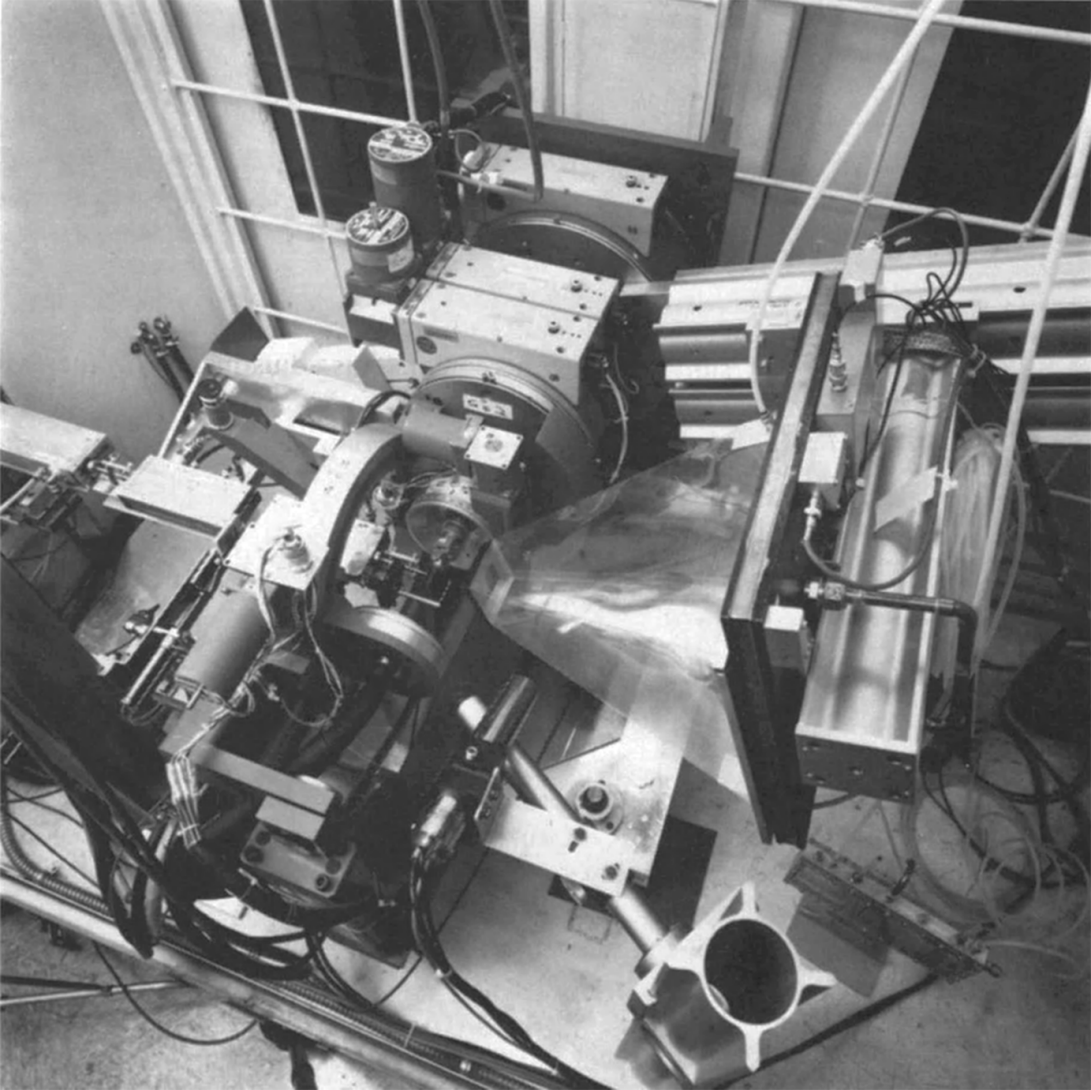
Photograph of the first MWPC area detector for MX studies (on the right, viewed from the top) and associated fast X-ray shutter, ion chambers, goniometer and other equipment installed on SSRL BL1-5 in the rear hutch *ca*. mid-1980s optimized for MX MAD data collection.

More specifically, at SSRL in the late 1970s, we were motivated to replace film with improved detectors and chose a 2D area detector having resolution sufficient for protein crystallography on relatively small unit cells, high detection efficiency and a digital readout. An ideal candidate at the time was a multiwire proportional counter (MWPC) detector which had been recently adapted and characterized for MX by Ng. H. Xuong, Ron Hamlin and Chris Nielsen at UCSD and V. Perez-Mendez at LBNL and their colleagues (Cork *et al.*, 1974 ▸). Paul Phizackerley, whom I had met earlier while we were both postdocs with Jack Dunitz at the ETH, had by then joined SSRL to lead the MX program in our SMB center. Paul, together with Carl Cork, collaborated with the UCSD and LBNL teams to integrate such a MWPC detector into SSRL’s BL1-5 in the rear hutch which was being optimized for MAD MX studies (Phizackerley *et al.*, 1986 ▸). This beamline was used for MAD phasing in the solution of the basic blue cucumber protein structure described above in Section 2.3. The front hutch on BL1-5 was configured for single-crystal diffractometry studies using a modified Enraf-Nonius CAD-4 diffractometer (Phillips *et al.*, 1979 ▸) and was used for, among other things, the accurate measurements of anomalous dispersion terms also described in Section 2.3. During the 1980s, this tandem two-instrument arrangement on BL1-5 was SSRL’s flagship facility for X-ray MX diffraction studies. Fig. 2 ▸ shows a photograph of the BL1-5 endstation instrumentation as configured for MAD MX studies as it appeared in the 1980s.

Around the same time, it is important to note that a parallel and conceptually similar development was being pursued in France at the LURE synchrotron facility by Roger Fourme together with George Charpak and collaborators (Kahn *et al.*, 1980 ▸; Fourme *et al.*, 1995 ▸). Their approach used a spherical drift multiwire proportional chamber and also proved to be very successful in recording high quality MX data, including applications to MAD phasing. There was frequent and excellent communication between the LURE and SSRL teams in the late 1970s into the 1980s as these exciting detector developments evolved on different sides of the Atlantic.

### 3.2. Cryo-cooling, robotics, automation and remote access

A second transformational R&D and instrumentation development at SSRL and other synchrotron biology centers worldwide was that of complete automation of MX beamlines, including cryo-cooling and remote access.

While the benefits of cryo-cooling protein crystals to mitigate X-ray-induced damage during X-ray irradiation were first demonstrated in 1969, the method did not gain widespread adoption in macromolecular crystallography until the early 1990s (Haas, 2020 ▸). In collaboration with Håkon Hope at UC Davis, SSRL SMB team members built a version of Hope’s heater-generated gas cold stream device (Bellamy *et al.*, 1994 ▸), installed it on BL7-1 and further refined the mounting and flash-cooling tools that he had pioneered (Parkin & Hope, 1998 ▸).

Key innovations included localized heating, and an on-axis geometry aligned with the beamline goniometer’s rotation axis, which together reduced turbulence and icing of the sample. This aerodynamic configuration in addition tolerated variations in sample length better, and remains in use at SSRL today for its reliability. Equally important was the development of a practical, user-accessible toolkit for cryogenic sample handling, supported by comprehensive online documentation that enabled the crystallographic community to readily adopt these methods. The SMB team played a central role in refining the sample mounting bases and specialized cryo-tools, simplifying the safe transfer and manipulation of crystals at cryogenic temperatures. Many of these tools, or derivatives, were later commercialized by companies such as Blake Industries, Crystal Positioning Systems and Hampton Research.

These manual cryogenic sample handling methods directly inspired and informed the design of the Stanford Automated Mounter (SAM) robot. The robot’s grippers and dumbbell magnet tool were explicitly modeled on the cryo-tools originally developed in the early 1990s, underscoring the lasting impact of these early innovations.

For us, these developments were motivated beginning in the 1990s, in part by the need for users to screen large numbers of crystals at cryogenic temperatures. This was the case in particular for an ambitious project to solve the structure of RNA polymerase that Roger Kornberg and collaborators at Stanford were working on at that time (Cramer *et al.*, 2000 ▸). Finding high diffraction quality crystals for this large macromolecular assembly was challenging and screening large numbers of crystals turned out to be essential. The manual process at the time was slow and tedious and it was very clear that a suitably designed sample handling robot could be exceptionally valuable and enabling. A second factor was that we received supplemental funding to our NIH center grant to develop a ‘collaboratory’ and part of that project was to further develop automation and remote access for our MX beamlines. A third factor was our involvement in the Joint Center for Structural Genomics (JCSG), one of the NIH-funded structural genomics centers in the US. Here again the need for automation was key, including the robotics, but going beyond to deliver full automation in a structure determination pipeline. Structural genomics at SSRL is briefly discussed later in Section 4.

**Figure 3:**
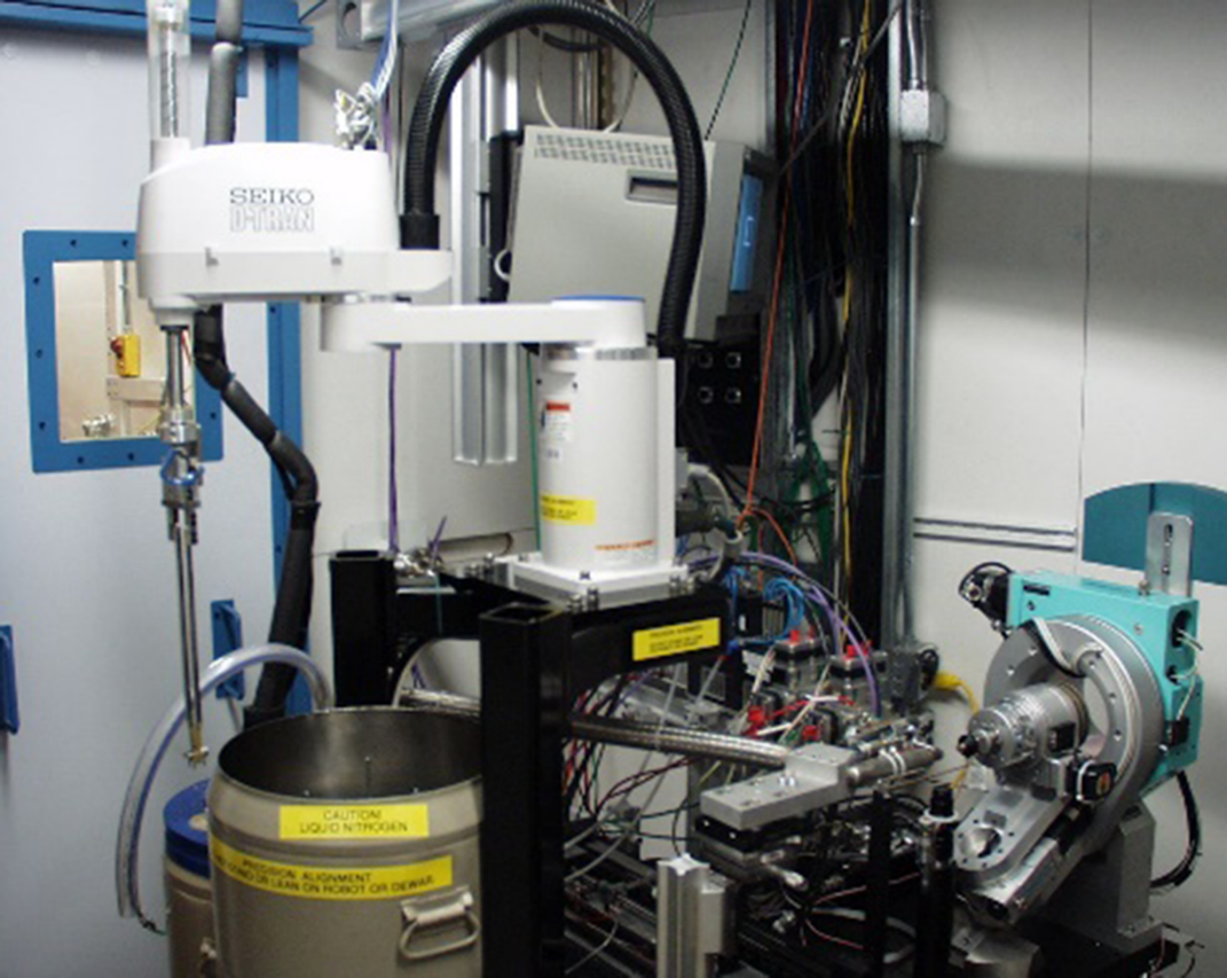
Photograph of the first robot installed on SSRL BL7-1 *ca*. early 2000s. The robot mimicked many of the manual operations developed for manual cryocooling and became the basis for fully automated sample handing under cryo-conditions.

The SSRL SMB MX team embarked on the development of a compact robotic system for mounting crystals under liquid nitrogen temperatures. This robot was designed to be fully integrated into the diffraction instrumentation and control system (Cohen *et al.*, 2002 ▸). The first robot prototype system was installed and commissioned on SSRL BL11-1 in June of 2001. The system (later called SAM for Stanford Auto-Mounter) was then installed with improvements on three additional SSRL MX beamlines (1-5, 9-1, 9-2) in 2002 and on the fourth MX beamline (11-3) when it was commissioned in 2003. A photograph of the SAM system installed on BL7-1 is given in Fig. 3 ▸. The SAM system proved to be exceptionally reliable and efficient, with the entire fully automated mounting operation taking about 80 s and the demounting operation about 50 s.

The SAM system was a key element of SSRL’s goal to develop a robust integrated approach for MX experiments with cryogenically preserved samples, which included remote-access automated crystal screening, data collection and data processing. Hardware components on the beamlines requiring movement were all mechanized and encoded, and along with other devices (*e.g.*X-ray optics, goniometer, video cameras, robot, detector, *etc*.) were controlled or monitored through an integrated instrument control system software package *DCS/Blu-Ice* (McPhillips *et al.*, 2002 ▸). This software and hardware infrastructure was able to be accessed fully remotely by users beginning in 2005. By 2007, ∼75% of SSRL’s MX user groups were doing so and collecting data remotely. This has steadily grown to be greater than 95% today. It is also worth noting that all MX beamlines at SSRL utilize the standardized*Blu-Ice* user interface, facilitating user training and interoperability as well as beamline support. The *Blu-Ice* interface has also been adapted to control the SMB bioSAXS/WAXS BL4-2.

A more detailed accounting of this new paradigm for MX at SSRL, which I believe was the first fully remote-access MX user program, is described in the paper by Soltis *et al.* (Soltis *et al.*, 2008 ▸). Upgraded versions of the SAM system are still in operation with increased speed and efficiency (Russi *et al.*, 2016 ▸) and in 2020 began supporting remote-access data collection using samples at non-cryogenic temperatures and controlled humidity conditions at SSRL’s BL12-1, thus facilitating room temperature static and time-resolved MX in a remote and high throughput manner. Many of the developments in robotics and automation on the MX beamlines have also been implemented on XAS and SAXS/WAXS and other beamlines at SSRL. It is again important to note that similar developments were occurring at other synchrotron facilities with SMB centers worldwide and some of these are described in the other articles in this special issue of *Journal of Synchrotron Radiation*.

## 4. Structural genomics and the PDB

In the early 2000s, a new initiative in structural genomics was gaining momentum and support worldwide. In the US, the NIH National Institute of General Medical Sciences (NIGMS) under the leadership of its then Director Marvin Cassman, started the Protein Structure Initiative whose goal was to fund a group of what ultimately became 12 centers that aimed to develop new high-throughput methods and determine thousands of novel uncharacterized protein structures, dramatically advancing knowledge of protein structure and function. Synchrotron MX was by far the most prolific method and extensive use of recombinant methods including tagging were a key part of the high throughput protein production and purification pipelines. We at SSRL were very fortunate to be able to partner with Ian Wilson and colleagues at The Scripps Research Institute (Ian Wilson was also the PI), John Wooley and colleagues at UCSD and the Sanford-Burnham Institute and others to create the Joint Center for Structural Genomics (JCSG), which began operation in 2000 (Elsliger *et al.*, 2010 ▸). SSRL hosted the Structure Determination Core for the JCSG over the 15 years of its existence. JCSG used the SSRL beamlines to very successfully solve around 1600 new structures which were deposited in the Protein Data Bank (PDB; Berman *et al.*, 2000 ▸). As noted in the comprehensive perspective on structural genomics programs worldwide by Michalska & Joachimiak (2021 ▸), the outcome over all the programs internationally was more than 9400 new structures of which ∼90% were determined by crystallography and the balance by NMR. Synchrotron radiation played an essential enabling role as the centers’ high throughput pipelines incorporated instruments at synchrotron beamlines and the majority of the X-ray structures were determined by these resources.

The PDB (Berman *et al.*, 2000 ▸) is a well known and invaluable resource for structural information on macromolecules and can be interrogated for information that demonstrates the impact of SR on MX. In 1994, about 1% of MX deposits were of structures done with synchrotrons and by 1999 this had risen to 50%. Since 2016, more than 90% of the deposits each year have utilized SR. As of the end of 2024, there were more than 165000 synchrotron-based structures. As is also well known, but important to emphasize again here, artificial intelligence program systems for structure prediction like *AlphaFold* developed by Google’s DeepMind (Jumper *et al.*, 2021 ▸) utilize the PDB for training and validation. I think it is fair to say that synchrotron-radiation-enabled MX has been crucial in the development and success of these modern computational predictive methods and will remain an essential source of experimental data on macromolecular structures and functions into the foreseeable future.

## 5. Additional perspectives and looking toward the future

Looking back on the evolution of the synchrotron structural biology enterprise over these past years, one can recognize several important characteristics and trends that have led to what is now more than 50 years of success, growth and impact, and that together create a platform for future success. These of course include foremost the people; in particular, the many dedicated scientific, technical and administrative staff who develop the instruments and techniques on the synchrotron beamlines, train and support the users. Workforce development is essential now and into the future. Sustainable funding is another key factor. In the US, for example, this has been primarily provided by the NIH, initially through its Division of Research Resources, then the National Center for Research Resources (NCRR) and currently the National Institute of General Medical Sciences (NIGMS) through its P30 ‘Mature Synchrotron Resources (MSRs) for Structural Biology’ grant program. Center support has also been provided by the DOE through its Office of Biological and Environmental Research (BER) where appropriate to its missions, and in a few specific cases by private foundations and industry, typically for specialized beamlines and instrumentation. Maintaining high priority for core operations and R&D funding and seeking means to diversify support are important objectives for the future.

Another essential and important element specifically highlighted above in Section 3 is the R&D programs which have led to new innovations in synchrotron-based technologies and methodologies. Collaborative research between the centers and their scientific and technical staff with the user community, often involving challenging science projects, drives the frontiers of the technologies while contributing to solutions of very challenging biological problems. The evolved and new technologies are then made available to the general user community so as to further enable scientific discovery. In the future, expanded collaborations among the structural biology synchrotron centers nationally and internationally and sharing in the development of new technologies will benefit the user community and further expand the capabilities for scientific discovery.

Training and dissemination have also been essential for growing the biological sciences SR user community. Innovations like beamline automation and remote access have contributed to engaging the broader community, educating and attracting scientists who are not experts but who can make use of the synchrotron techniques in solving their problems. This has grown a ‘non-traditional user base’. Extensive training, including local and remote workshops, is essential in training new users and growing the next-generation talent. For example, SSRL, in partnership with LCLS, sponsors an annual user meeting (in person, but with hybrid access for some sessions) that typically has ∼500 on-site participants attending the plenary sessions and associated satellite workshops. In a typical year, SMB staff also offer topical workshops, both on-site and in remote locations, to train users in the techniques available and carry out experiments remotely. In the future, expanded cooperation among the centers and coordination of training using common modules will be more effective and efficient. The synchrotron structural biology centers also have a strong program in outreach and dissemination of technical and scheduling information and scientific outcomes through a variety of social media and web-based tools. As with technology development and training and outreach, coordination among centers can better reach the broader scientific community and most effectively engage and grow the user base.

The dramatic evolution of SR source technology and performance that has occurred over these past 50 years is another remarkable and impactful story, which I can only briefly mention here, while noting that these developments have made possible increasingly challenging experiments in synchrotron-enabled structural biology. Through the skill and creativity of accelerator scientists, there has been a remarkable evolution from the early parasitic storage-ring-based sources like SSRP (the first generation sources), to dedicated storage ring sources like NSLS at Brookhaven National Laboratory and the Photon Factory in Japan at KEK (second generation sources), to SPEAR3 – the upgrade of SPEAR to an early low-emittance third generation source, and then on to dedicated high brightness storage ring sources with undulators as the main X-ray device such as ESRF, APS and SPring-8 and many other third generation sources. It is on these third generation sources, a number of which are described in the articles in this special virtual issue of *Journal of Synchrotron Radiation*, that the majority of the structural biology research is done today. An excellent perspective of the generations of storage ring sources and their increases in brightness in the context of MX, particularly in Europe, is found in the 2011 British Crystallographic Association Lonsdale Lecture by John Helliwell (Helliwell, 2011 ▸). A more technical description of the source characteristics of storage-ring-based synchrotron sources and production of X-rays by insertion devices is found in the comprehensive overview article by Kwang-Je Kim (Kim, 1995 ▸).

In the 1990s, accelerator physicists proposed that an innovative X-ray free electron laser could be built from a linac-based electron source (fourth generation sources) (Pellegrini, 1992 ▸). LCLS at SLAC was the first one of these machines to be built and it demonstrated first X-ray lasing in 2009 (Emma, 2010 ▸) and rapidly thereafter became operational. The scientific case for LCLS was in part motivated by structural biology experiments that would take advantage of the ‘diffraction before destruction’ paradigm proposed by Janos Hajdu, Richard Neutze and collaborators (Neutze *et al.*, 2000 ▸). The suite of first instruments on the LCLS included two (the X-ray pump probe station and the coherent imaging station) that were successfully used for early MX studies. The realization of the LCLS, its characteristics and scientific productivity and impact are described in detail in the accompanying article in this special virtual issue by Mous, Hunter, Poitevin, Boutet and Gee (Mous *et al.*, 2025). SSRL’s SMB scientific and technical staff worked closely with their LCLS counterparts to develop and deploy fixed target methods for MX, initially using modified instrumentation on the LCLS X-ray pump probe station (XPP) and subsequently on the dedicated MX station, MFX. SSRL’s MX undulator beamlines (BL12-1 and 12-2) provide a test bed for new methodologies and staging of experiments prior to their execution at LCLS. XFELs subsequently became operational in 2011 in Japan (SACLA) and in 2017 in Germany (European XFEL) and then in Korea and Switzerland. An overview of the European XFEL can be found in another article in this special issue (Oberthür *et al.*, 2025) which also includes a perspective on the interesting and remarkable evolution of the unique scientific environment (or ecosystem) in and around the DESY campus in Hamburg. Developments at the SACLA XFEL in Japan are also described in another comprehensive article in this special edition of *Journal of Synchrotron Radiation* (Yamamoto & Kumasaka, 2025).

While the scientific impact of synchrotron-enabled MX can be shown by metrics such as those mentioned above from the PDB and others, perhaps one of the most striking demonstrations of its impact is seen in its very significant or partial contributions to seven Nobel prizes that have been awarded since 1997. The first to make extensive use of synchrotron MX was Sir John Walker in 1997 for his work on F1-ATP synthase. In 2003, the Nobel prize was awarded to Roderick MacKinnon and Peter Agre for their research on potassium ion channels, and in 2006 to Roger Kornberg for his studies on RNA Polymerase II. The 2009 Nobel prize was shared by Venke Ramakrishnan, Tom Steitz and Ada Yonath for their research on the ribosome. In 2012, the Nobel prize was awarded to Brian Kobilka and Robert Lefkowitz for work on G protein-coupled receptors. Most recently, the 2018 Nobel prize was awarded to Frances Arnold for her research on directed evolution and in 2020 to Jennifer Doudna and Emmanuelle Charpentier for their work on CRISPR.

One might also include in this accounting a mention of the most recent 2024 Nobel prize awarded to Demis Hassabis and John Jumper (also shared with David Baker) for their work on AlphaFold2 (Jumper *et al.*, 2021 ▸) whose prediction of macromolecular structures depended on PDB depositions, a very large number of which came from synchrotron-based determinations. Synchrotron MX will continue to be essential for high resolution structure determination of large multicomponent systems and their interactions and validation of structures predicted from ML algorithms like AlphaFold2 and its variants. For certain, static and dynamic studies of biomolecular structure well into the future will involve both experimental measurements and computational modeling, while tackling the most challenging problems and yielding remarkable insights into some of the most important chemical processes in biology.

Another area where synchrotron-enabled MX has had a significant contribution is in helping accelerate the discovery of new therapeutic approaches during critical health crises like pandemics. For example, during the early stage of the COVID-19 pandemic in Spring of 2020, SSRL’s MX microfocus undulator beamlines were able to play an important role in rapidly enabling research on COVID-related problems. SSRL’s MX user community focused on three central pillars of COVID-related structural biology: (1) determining high resolution structures of SARS-CoV-2 proteins and their binding partners, including time-resolved measurements, to study mechanisms of interaction; (2) characterizing neutralizing antibodies and nanobodies bound to the viral Spike protein to inform vaccine and biologics design; and (3) investigating viral protein complexes with small molecules, inhibitors and lead compounds to accelerate drug discovery. SSRL’s MX beamlines supported 70 COVID-related research proposals mainly from across the US and Canada, resulting in ∼230 new structures being deposited in the PDB. These efforts resulted in ∼70 peer-reviewed publications contributing to the development of 50 new potential drugs and vaccines, 10 of which are currently in various stages of clinical trials. It should be noted that other synchrotron structural biology centers in the US and worldwide played similar roles in the COVID-19 pandemic response.

Electron-based imaging methods for biological studies have experienced a renaissance in the past 10–15 years. There has been dramatic worldwide growth in the capabilities and utilization of cryogenic electron microscopy (CryoEM). While this is a technique whose principles have been known for some time, transformational hardware developments, including advanced direct electron detectors, brighter electron sources and more stable cryo-sample stages, together with advances in software and computational algorithms for automatic data collection and analysis, have led to remarkable advances in the determination of increasingly high resolution structures of macromolecules with heterogeneous conformations and compositions. The related technique of cryogenic electron tomography (CryoET) has also seen significant growth with its ability to image complex macromolecular assemblies and cellular components *in situ* ranging from nanometre to sub-nanometre resolutions. While CryoEM/ET instruments can be located in individual PI’s labs, a more efficient operational model, similar to that for synchrotron beamlines, has also emerged. This involves colocation of multiple instruments at national or regional centers, some of which are themselves co-located with synchrotron structural biology facilities. At SLAC within the SSRL Directorate, for example, under the leadership of Wah Chiu, there are two NIH-funded national centers, one for CryoEM and another related to CryoET. In addition to sharing support services like user process management and scheduling and ES&H resources, there is real opportunity for multi-technique applications as well as synergies in areas of R&D such as freeze trapping of intermediates for structural studies and the increasing development and applications of AI/ML data analysis algorithms.

As we look at recent trends and innovations in structural biology which are complementary to traditional MX, and project toward the future, those including computational modeling and simulations, the advent of X-ray free electron lasers, applications of AI/ML, and electron-based imaging all stand out in my opinion. There is strong synergy among all of these methodologies with synchrotron structural biology and, together with other methodologies, we see that integrated multimodal research across multiple length- and time-scales is increasingly becoming the paradigm for gaining detailed insights into complex biological structure and function. For example, the powerful combination of CryoEM and high resolution MX structural data is beautifully illustrated in the structural determination of the nuclear pore complex by Hoelz and collaborators (Bley *et al.*, 2022 ▸; Petrovic *et al.*, 2022 ▸), a channel comprising ∼1000 protein subunits embedded in the nuclear envelope that regulates the transport of macromolecules between the eukaryotic cell’s nucleus and cytoplasm.

**Figure 4:**
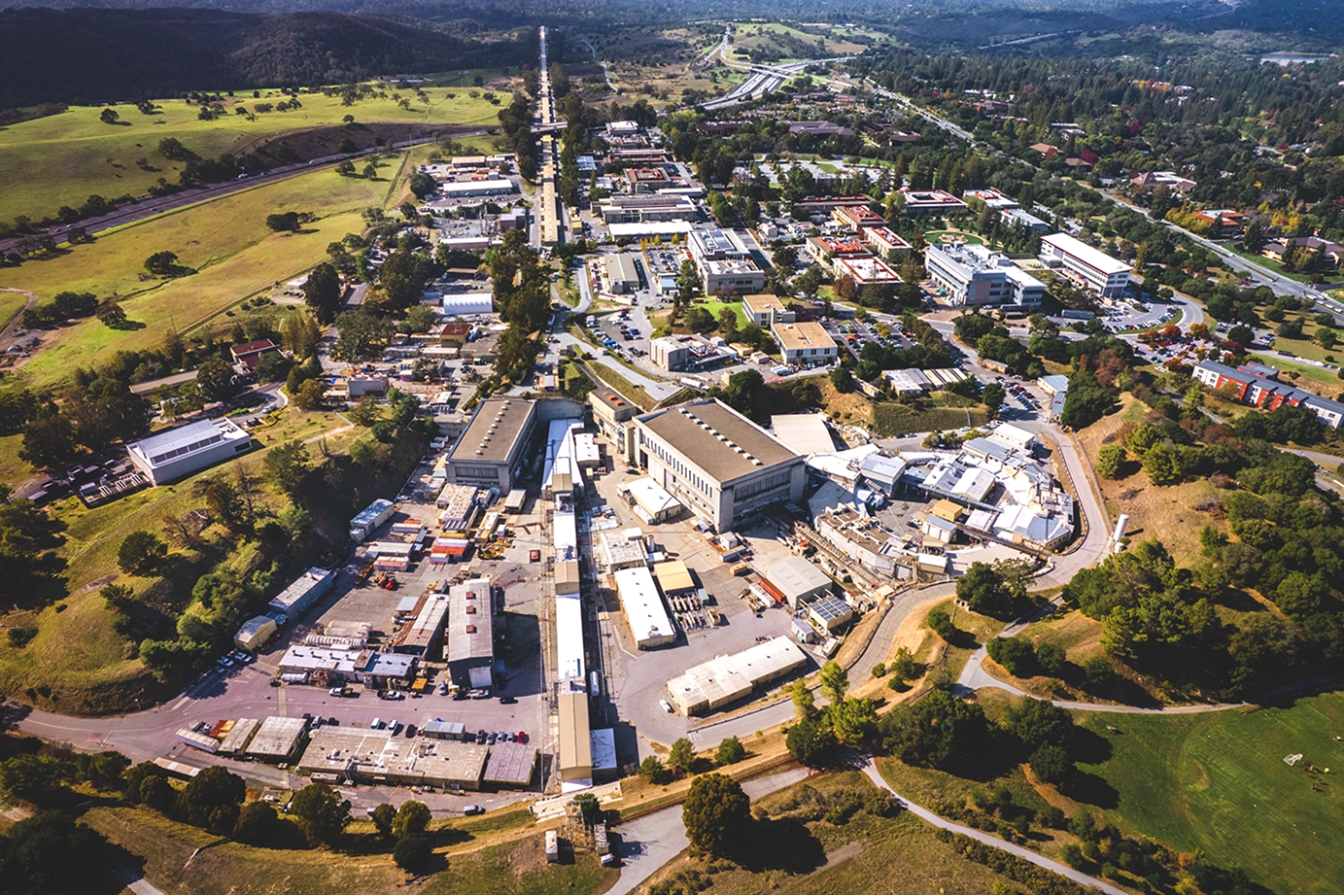
Aerial view of the SLAC campus. The last part of the 3 km SLAC linac, which begins at the top of the photograph and ends in the research yard, is visible running vertically down from the top. The SSRL complex and its SPEAR3 storage ring is seen in the right middle. The LCLS electron beam transits the research yard from the end of the linac and enters the hill seen at the bottom of the photograph, with the experimental halls for the LCLS off of the bottom. The CryoEM facilities are located in a building near SSRL and in one on the central part of the main quad in the upper right. Photograph courtesy of Olivier Bonin/SLAC National Accelerator Laboratory.

Today at SLAC we have a constellation of structural techniques based on X-rays from synchrotron and free electron laser sources and on electrons for studying and interrogating biological systems – ranging from at the scale of atoms and even electrons all the way up through complex multiprotein complexes and molecular machines, to cellular structures and organelles. An aerial view of the SLAC campus can be seen in Fig. 4 ▸. Further, we can study structural dynamics on time scales from minutes to as short as sub-femtoseconds. When combining these complementary X-ray- and electron-based structural techniques with other data, one can begin to gain a true structural and functional knowledge of behavior in very complicated biological systems. Further, SLAC is located on Stanford land, close to the main academic center of the University where many faculty in the Schools of Medicine, Engineering and Humanities and Sciences have active R&D programs which can utilize and help drive the development of synchrotron-based techniques for the study of structure and function of biomolecules. Located relatively nearby are also leading biotech and biopharma companies and research institutes including the Universities of California at Berkeley and at San Francisco and National Laboratories at Berkeley and Livermore. This ecosystem fosters innovation and scientific discovery at the forefront of studying biological structure and function while offering unique research opportunities to students and postdocs and training the next generation.

## 6. Conclusion

Much more than was probably expected 50 years ago has been achieved and I think it can be said that structural biology was transformed by the advent of synchrotron-enabled experiments. I expect that in the future we will see stronger partnerships, collaborations and coordination among the structural biology synchrotron centers, and of those centers with CryoEM/ET centers and other university, national lab and industry research initiatives. Development of new experimental and computational AI/ML approaches will in combination enable the user community to push the boundaries of discovery in space and time, contributing to our fundamental understanding of biological structure and function and benefitting areas that include human health, bio-energy and bio-sustainability. It is a privilege that SSRL has been part of this grand venture since the early 1970s and continues together with LCLS and CryoEM/ET at SLAC, on a path toward a vibrant future for the coming decades.

This special virtual edition of *Journal of Synchrotron Radiation* is remarkable in that it celebrates 50 years of achievements in structural biology since those pioneering experiments in the early 1970s. We all owe many thanks to John Helliwell and the Editors of the journal whose idea and commitment has given rise to this special edition. Of course, it is the many hundreds of PI’s and their research groups and students around the world who have done the outstanding science that has continued to fuel the success of this endeavor. In closing, I would also again like to recognize and thank the scientists who have contributed many other articles in this special virtual issue, describing structural biology activities and innovative developments at many of the major synchrotron centers around the world.
